# Comparative Skin Transcriptomics Reveals Key Regulators of Cashmere Fiber Production in Inner Mongolian Goats

**DOI:** 10.3390/ani16060927

**Published:** 2026-03-16

**Authors:** Hafiza Arooba Riaz, Muhammad Irfan Khan, Kiran Zahra, Rahmat Ali, Dejun Ji

**Affiliations:** College of Animal Sciences and Technology, Yangzhou University, Yangzhou 225009, Chinamh23052@stu.yzu.edu.cn (M.I.K.);

**Keywords:** cashmere goat, secondary hair follicle, transcriptome sequencing, hair follicle development, differential gene expression, fiber formation

## Abstract

Cashmere goats produce high-value fine fibers derived from secondary hair follicles; however, the molecular mechanisms underlying this trait remain incompletely understood. In this study, comparative transcriptome sequencing was performed on skin tissues from Inner Mongolian cashmere goats and normal goats to characterize gene expression differences associated with cashmere fiber production. High-quality RNA-seq data with strong mapping efficiency and reproducibility were obtained across all samples. Differential expression analysis identified 1543 significantly differentially expressed genes (DEGs) between cashmere and normal goats, including genes involved in hair follicle morphogenesis, epidermal differentiation, cell proliferation, and extracellular matrix organization. Multivariate analyses showed a clear transcriptomic separation between fleece types, indicating that fleece phenotype is the primary driver of variation in global gene expression. Functional enrichment revealed significant involvement of the Wnt, MAPK, and PI3K–Akt signaling pathways, and several biologically relevant regulators of hair follicle development and hair cycle control, including *FGF5*, *SOX9*, *LHX2*, and *VDR*, were differentially expressed. Gene fusion events were rare and showed no group specific patterns, whereas alternative splicing was widespread, with exon skipping as the predominant splicing event in goat skin. Overall, these results provide quantitative transcriptomic evidence linking signaling regulation, follicle development, and structural differentiation to secondary hair follicle activity and cashmere fiber formation, offering candidate genes and molecular pathways for functional validation and molecular breeding in cashmere goats.

## Simple Summary

Cashmere fiber is a valuable natural textile produced by specialized secondary hair follicles in cashmere goats. Improving fiber yield and quality requires a better understanding of the biological mechanisms that distinguish cashmere producing goats from normal goats. In this study, we compared the skin transcriptomes of Inner Mongolian cashmere goats and normal goats using RNA sequencing to identify genes and pathways associated with fleece type. We detected 1543 differentially expressed genes enriched in processes related to hair follicle development, epidermal differentiation, and extracellular matrix organization. Key signaling pathways, including Wnt, MAPK, and PI3K–Akt, were significantly involved, highlighting their importance in regulating secondary hair follicle activity. We also found that alternative splicing, particularly exon skipping, is a common regulatory mechanism in goat skin. These findings improve our understanding of the molecular basis of cashmere fiber formation and provide candidate genes for future functional research and breeding programs aimed at enhancing economically important fleece traits in cashmere goats.

## 1. Introduction

Cashmere is a premium natural fiber produced by cashmere goats (*Capra hircus*) and is valued for its fineness, softness, and thermal insulation. Cashmere production supports livelihoods across pastoral regions, and the global value chain has become increasingly concentrated in China, reflecting the country’s major role in raw cashmere production and processing [1]. Among Chinese breeds, the Inner Mongolian cashmere goat is a well-recognized genetic resource and is widely regarded for favorable fleece traits, including cashmere yield and fiber quality [2]. Improving cashmere yield and fiber diameter while maintaining animal adaptability remains a priority for breeding programs and for sustainable industry development [2].

From a biological perspective, cashmere fibers arise from a “double-coat” system formed by two follicle types. Primary hair follicles (PHFs) generate coarse guard hairs for protection, whereas secondary hair follicles (SHFs) produce the fine undercoat (cashmere) that provides insulation. Consequently, SHF density, SHF/PHF ratio, and SHF cycling activity are among the most important determinants of cashmere yield and fiber quality. Hair follicles are dynamic mini-organs that repeatedly undergo cyclic regeneration throughout life. The classical cycle comprises anagen (active growth), catagen (regression), and telogen (relative rest), coordinated by interactions between epidermal stem/progenitor compartments and dermal signaling niches such as the dermal papilla [3,4,5]. In cashmere goats, follicle cycling exhibits pronounced seasonality and synchrony across the body, making them a valuable model for understanding environmental control of follicle activity and for identifying molecular regulators of fiber production [6,7,8].

At the molecular level, hair follicle morphogenesis and cycling are controlled by conserved signaling pathways and transcriptional networks. Canonical developmental pathways, including Wnt/β-catenin, BMP/TGF-β, Hedgehog, and Notch, coordinate epidermal lineage decisions, stem cell activation, and hair shaft differentiation [4,5,9]. Additional signaling systems, such as MAPK and PI3K–Akt, integrate growth factor cues and cellular metabolism with proliferation and survival programs in follicular keratinocytes and dermal cells [9]. Functional studies in mammalian models have also highlighted the importance of specific regulators of hair cycle progression and hair length. For example, fibroblast growth factor 5 (*FGF5*) acts as a key negative regulator of hair elongation and promotes the anagen to catagen transition; targeted and spontaneous mutations in *FGF5* produce long hair phenotypes across mammals [10]. Similarly, cell–cell adhesion and desmosomal integrity are crucial for follicular differentiation and hair shaft structure; defects in desmosomal cadherins, such as DSG4, cause hypotrichosis and hair shaft abnormalities [11]. These examples illustrate how changes in signaling, transcriptional regulation, and structural differentiation can translate into measurable fiber traits.

With the expansion of high-throughput sequencing, transcriptomic profiling has become a central approach for dissecting the genetic architecture of hair follicle development in livestock. Early RNA-seq studies in cashmere goats focused on comparing anagen, catagen, and telogen stages, identifying hundreds to thousands of differentially expressed genes (DEGs) linked to follicle cycling and implicating pathways such as Wnt, TGF-β, Shh, and Notch in stage transitions [12]. Subsequent work expanded toward understanding seasonal biology and photoperiod regulation. For instance, skin transcriptome profiling across multiple time points under natural versus shortened photoperiod conditions identified extensive periodic gene expression and large DEG sets during hair cycle transitions, supporting the concept that environmental cues reshape gene regulatory programs in goat skin [7]. Comparative seasonal transcriptome studies across goat types have also reported expression differences associated with yearly cycling and between cashmere and non-cashmere phenotypes, further emphasizing the biological complexity of skin and follicle regulation across breeds and environments [7].

In parallel, developmental studies have examined SHF formation in fetal skin, which is critical because SHF establishment and maturation strongly influence adult cashmere traits. Integrated mRNA–miRNA analyses across fetal stages in Inner Mongolian cashmere goats have provided evidence that coordinated transcriptional and post-transcriptional regulation contributes to SHF development, identifying candidate regulatory axes and signaling components relevant to follicle morphogenesis [6]. Additional transcriptome work on isolated follicles has improved mechanistic interpretation by reducing cellular heterogeneity inherent to bulk skin sampling; RNA-seq analyses of isolated hair follicles during anagen and catagen have characterized follicle-intrinsic DEGs and pathway changes associated with regression programs [13]. Comparative transcriptome analyses of fetal skin have further identified key genes associated with hair follicle morphogenesis and development in cashmere goats [14]. More recently, single-cell transcriptomic atlases of cashmere goat hair follicle morphogenesis and regeneration have mapped the cellular heterogeneity of epidermal and dermal compartments, including dermal papilla states, and highlighted lineage trajectories and regulatory markers that are difficult to resolve by bulk RNA-seq alone [15,16]. Collectively, these studies demonstrate rapid progress in profiling follicle biology in cashmere goats and provide valuable resources for candidate gene discovery.

Despite these advances, key knowledge gaps remain. First, many transcriptome studies have focused on seasonal cycling in cashmere goats, developmental time courses, or comparisons of isolated follicles. While informative, these designs do not always resolve the core transcriptomic differences that distinguish cashmere-producing skin from normal goat skin under comparable sampling and analytical conditions. Second, bulk skin transcriptomes can be influenced by multiple cell types (epidermal, dermal, immune, vascular), meaning that identifying robust fleece type signatures requires careful comparative analysis and validation of biological plausibility. Third, beyond differential gene expression, transcriptome regulation also includes features such as alternative splicing, which may contribute to follicle function and fiber traits but are less consistently evaluated in fleece-type comparisons. Therefore, direct comparative profiling between cashmere goats and non-cashmere goats, using standardized skin sampling and a unified RNA-seq pipeline, remains an important strategy to clarify fleece-type-associated molecular programs and to generate candidate genes for downstream functional studies and breeding.

To address these gaps, the present study performed comparative transcriptome profiling (RNA-seq) of skin tissues collected from Inner Mongolian cashmere goats and normal goats using a standardized biopsy procedure (as described in the Section 2). We systematically identify DEGs associated with fleece type, characterize functional enrichment patterns relevant to hair follicle development, epidermal differentiation, proliferation, and extracellular matrix organization, and evaluate transcriptome-level regulatory features including alternative splicing events. By integrating these analyses, our study provides a comprehensive view of gene expression differences associated with secondary hair follicle activity and cashmere fiber formation, thereby offering candidate genes and pathways for functional validation and molecular breeding strategies to improve economically important cashmere traits.

## 2. Materials and Methods

### 2.1. Ethics Statement

This study was supported by the Priority Academic Program Development of Jiangsu Higher Education Institutions (PAPD, 2014-134) and the Key Natural Science Research Project of Colleges and Universities in Jiangsu Province (21KJA230002). All experimental protocols were conducted in accordance with the *Guide for the Care and Use of Agricultural Animals in Research and Teaching* published by the Federation of Animal Science Societies (FASS, 2010). Animal experiments were approved by the Animal Care and Use Committee of the College of Veterinary Medicine, Yangzhou University (approval numbers: 202301011 and 202502008; approval dates: 11 October 2023 and 10 February 2025). All procedures complied with institutional animal welfare regulations to minimize animal discomfort and ensure humane treatment throughout the study.

### 2.2. Animal Selection and Skin Tissue Collection

**Figure 1:**
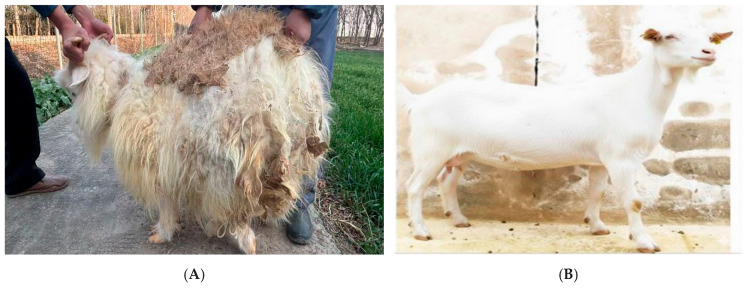
In the images, (**A**) Cashmere goats reveal a lush layer of soft, fine cashmere hidden beneath their guard hair, while (**B**) normal goats display only coarse primary fibers and a barely there undercoat.

Two groups of goats were selected for this study: normal (P) and Inner Mongolian cashmere goats (K) (Figure 1). The four normal goats (P1–P4) were aged 10–12 months. The four cashmere goats were divided into two age-defined subgroups: R3 and R4 (12 months) and C1 and C2 (15 months). All animals used in this study were sourced from a commercial goat breeding farm located in Gaoyou, Yangzhou City, Jiangsu Province, China. The goats were owned and managed by a local goat breeder under standard husbandry conditions. Sampling was conducted with the consent of the farm owner and in compliance with approved institutional ethical guidelines. Skin biopsy samples were obtained using standard veterinary dermatological procedures for transcriptomic studies of livestock skin [13]. A 5 cm × 5 cm hair patch was trimmed from the posterior edge of the left scapula near the midline, residual hair was removed with a razor, and the exposed skin was disinfected with alcohol and iodine. A sterile 1 cm circular biopsy punch was used to collect skin tissue, and Yunnan Baiyao powder was applied to control bleeding.

In total, four normal and four cashmere goat skin samples (1 cm^2^) were collected, immediately frozen in liquid nitrogen, and stored at −80 °C until RNA extraction for transcriptome sequencing.

### 2.3. RNA Extraction and Quality Control

Total RNA was extracted from approximately 30 mg of skin tissue using TRIzol™ reagent (Invitrogen RC112: Vazyme, Biotech Co., Ltd., Nanjing, China) according to the acid guanidinium thiocyanate–phenol–chloroform extraction method [17]. RNA purity and concentration were measured using a NanoDrop™ 2000 spectrophotometer (Thermo Fisher Scientific, Waltham, MA, USA) based on A260/A280 and A260/A230 absorbance ratios [18].

### 2.4. Library Construction and Transcriptome Sequencing

RNA-seq libraries were constructed using a poly(A)enriched, strand-specific library preparation protocol widely applied in transcriptomic studies [18]. Briefly, mRNA was enriched using oligo(dT) magnetic beads and fragmented into short fragments, followed by first strand cDNA synthesis using random primers. Second strand synthesis incorporated dUTP to enable strand specificity, after which cDNA fragments underwent end repair, A-tailing, adapter ligation, uracil DNA glycosylase digestion, and PCR amplification [19].

Sequencing libraries were converted into DNA nanoball (DNB) structures and sequenced on the BGI DNBSEQ platform with paired-end 150 bp reads (PE150) according to established high-throughput sequencing technology [20].

### 2.5. Bioinformatic Analysis

Sequencing data, also known as raw reads, underwent quality control (QC) to assess their suitability for analysis. SOAPnuke (ver. 2.3.1) [21] was used to filter out reads with adapter contamination, reads with more than 0.1% unknown base ‘N’ content, and low-quality reads [21]. The resulting clean data were then analyzed, visualized, and mined using the Online Multi-Omics Data Mining System.

Following QC, clean reads were aligned to reference sequences using HISAT2 (ver. 2.2.1) [22]. HISAT2 first anchors partial read sequences to the genome with global FM indexing, then uses local indexing to align the remaining sequences and extend the alignment region. After alignment, a second QC assessed alignment quality, including alignment rate and read distribution across reference sequences.

Gene quantification and expression analyses, including principal component analysis, correlation analysis, and differential gene screening, were performed. Clean data were aligned to the reference gene set using Bowtie2 (v2.3.4.3) [23]. Gene expression was quantified with RSEM (v1.3.1) [24], and clustering heatmaps were generated using pheatmap (v1.0.8) [25]. Differential gene detection was conducted using DESeq2 (v1.4.5) [26], DESeq2 [27] or PoissonDis [28] with thresholds of Q ≤ 0.05 or FDR ≤ 0.001.

Differentially expressed genes were further analyzed for gene ontology (GO) enrichment, pathway enrichment, clustering, protein-protein interaction networks, and transcription factors, using a Q-value threshold of ≤0.05. Gene fusion detection was performed with Ericscript (v0.5.5) [29] and alternative splicing events were identified using rMATS (v3.2.5) [30].

## 3. Results

### 3.1. Sequencing Data Quality and Preprocessing

High-throughput RNA sequencing was conducted on skin samples from eight goats, including four normal goats (P1–P4) and four cashmere goats categorized into two age groups (C1–C2: 15 months; R3–R4: 12 months). Quality assessment demonstrated consistently high-quality sequencing data across all samples. After rigorous filtering, an average of 22.07 million clean reads per sample was obtained, with a clean reads ratio ranging from 89.42% to 95.53%. The clean data were of high quality, as indicated by Q20 scores of at least 97.61% and Q30 scores of at least 91.06%, supporting the reliability of the sequencing results for subsequent analyses (Supplemental Table S1).

**Figure 2:**
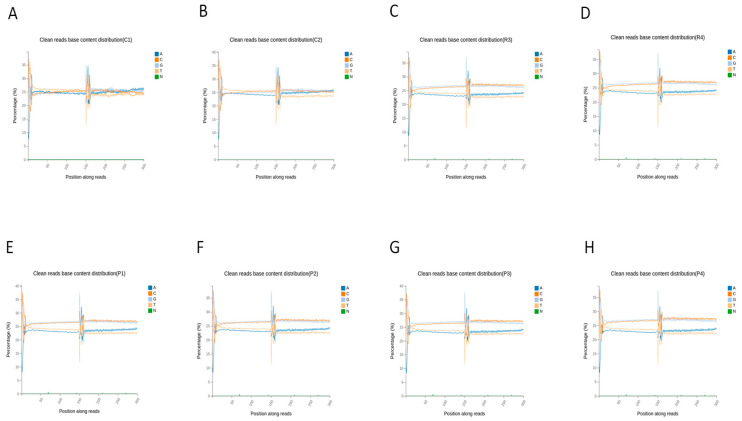
Base Content Distribution Graph of Clean Reads across all samples. (**A**) C1; (**B**) C2; (**C**) R3; (**D**) R4; (**E**) P1; (**F**) P2; (**G**) P3; (**H**) P4.

**Figure 3:**
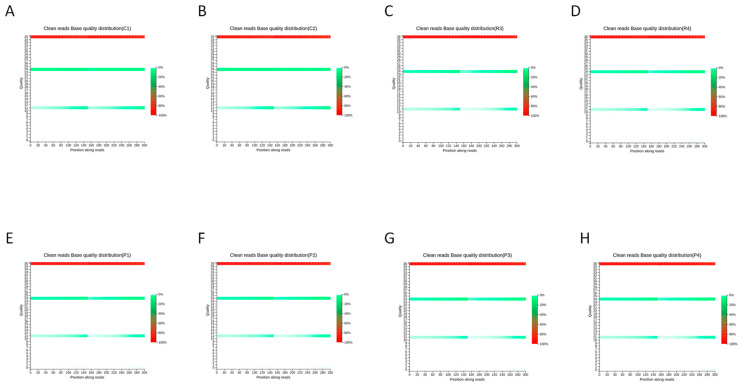
Base Quality Distribution Graph of Clean Reads across all samples. (**A**) C1; (**B**) C2; (**C**) R3; (**D**) R4; (**E**) P1; (**F**) P2; (**G**) P3; (**H**) P4.

The base content distribution indicates a balanced representation of nucleotides (A, T, C, G) across sequencing cycles in the clean reads, with no detectable bias. This finding confirms the absence of sequencing or adapter contamination artifacts (Figure 2). The base quality distribution shows that the median quality scores for all bases consistently exceed the Q30 threshold (accuracy > 99.9%), supporting the high base-calling accuracy of the sequencing data (Figure 3).

### 3.2. Efficient Mapping to Reference Genome and Transcript Coverage

Clean reads were successfully aligned to the goat reference genome. Mapping efficiency remained consistently high (Supplemental Table S2), with Total Mapping rates ranging from 96.09% to 98.98% and Uniquely Mapping rates from 91.73% to 93.11%. These results demonstrate that most sequencing data originated from the goat genome, with minimal ambiguous alignment.

**Figure 4:**
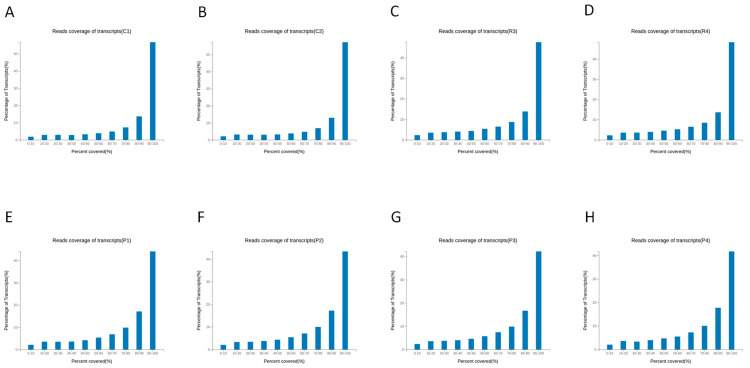
Uniform transcript coverage validates library preparation. (**A**) C1; (**B**) C2; (**C**) R3; (**D**) R4; (**E**) P1; (**F**) P2; (**G**) P3; (**H**) P4.

Subsequent alignment to the reference gene set (Supplemental Table S3) produced Total Mapping rates between 68.53% and 77.92% and Uniquely Mapping rates between 61.74% and 70.34%. These slightly lower percentages than in genome mapping are expected because this metric reflects the proportion of reads that map specifically to annotated exonic regions. Cashmere goat samples (C and R groups) consistently exhibited higher gene mapping rates than normal goats (P group), indicating potential differences in transcriptional activity or gene representation. Uniform read coverage was observed along the length of transcripts, without significant 5′ or 3′ bias (Figure 4). This uniformity confirms the integrity of the RNA samples and the suitability of the data for accurate gene expression quantification. Collectively, these quality control metrics indicate that the RNA-seq data are high-quality and suitable for comprehensive transcriptomic analysis.

### 3.3. Sample Correlation and Global Expression Patterns

**Figure 5:**
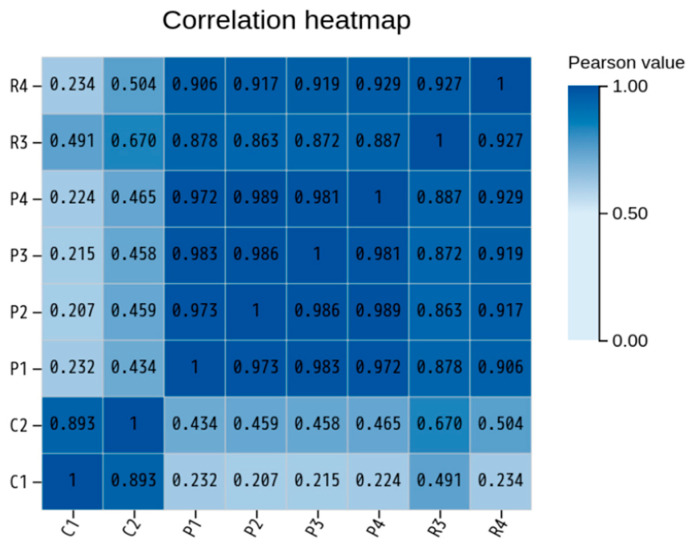
Correlation heatmap of gene expression among Cashmere (K) and normal (P) goat samples.

Pairwise correlation analysis of normalized gene expression values was performed to evaluate reproducibility and similarity among biological replicates. The resulting correlation heatmap demonstrated strong correlations within each group (K or P), with Pearson’s R values generally exceeding 0.90. In contrast, lower correlations were observed between the Cashmere and normal goat groups (Figure 5). These findings suggest high intra-group consistency and distinct inter-group differences.

**Figure 6:**
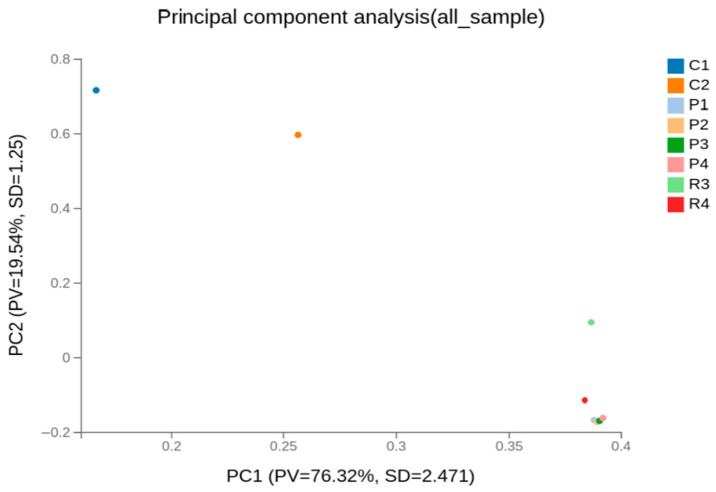
Principal component analysis (PCA) of global gene expression profiles.

Principal component analysis (PCA) provided additional support for these findings. The first two principal components explained 76.32% and 19.54% of the total variance, respectively, together accounting for 95.86% of the overall transcriptional variation (Figure 6). Minor variation within the Cashmere group was detected, potentially attributable to age-related or individual biological differences. Collectively, PCA revealed a pronounced global transcriptional divergence between Cashmere and normal goats.

Principal Component Analysis (PCA) plot of the global gene expression profiles. The clear separation of Cashmere goat samples (K) from Normal goat samples (P) along the first principal component (PC1) indicates that fleece type is the primary source of transcriptional variation.

### 3.4. Distribution of Gene Expression Levels

**Figure 7:**
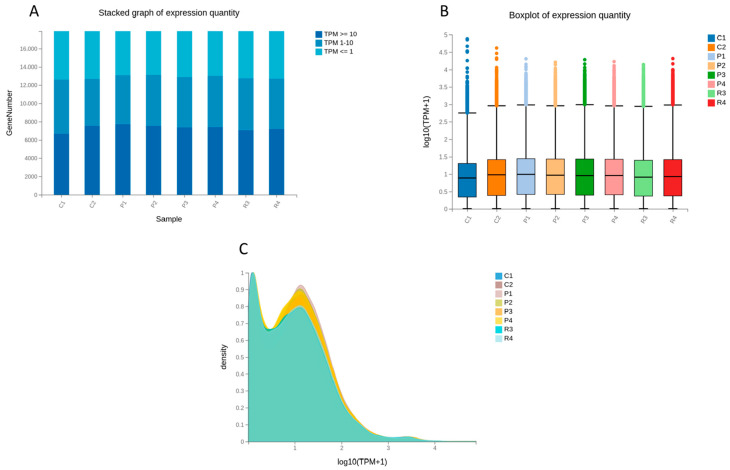
Distribution and comparison of gene expression levels across samples. (**A**) Stacked bar plot showing the number of genes at different TPM expression ranges. (**B**) Boxplot of log_10_ (TPM + 1) values for each sample. (**C**) Density distribution of log_10_ (TPM + 1) across samples.

Gene expression profiles and data quality were assessed by quantifying expression levels using TPM (Transcripts Per Million), with distributions summarized in Figure 7A–C. Stacked bar plots demonstrated that most genes were expressed in all eight samples. Most genes exhibited low to moderate expression levels (TPM 1–10), while a smaller subset showed high expression (TPM ≥ 10). The proportion of non-expressed genes (TPM ≤ 1) was also similar among samples. This uniformity suggests consistent sequencing depth and comparable transcript detection efficiency between normal and cashmere goats across age groups (Figure 7A). Boxplot analysis of log_10_ (TPM + 1) values showed similar median expression levels and interquartile ranges across samples, indicating effective normalization and minimal systematic bias (Figure 7B). Density plots of log_2_ (TPM + 1) further confirmed that global expression distributions were highly similar across samples, supporting the comparability of transcriptomic profiles between groups (Figure 7C).

### 3.5. Identification of Differentially Expressed Genes (DEGs)

Differential expression analysis was performed to identify genes associated with hair follicle traits specific to Cashmere goats. Genes were considered significantly differentially expressed if they met the criteria of log_2_ (K/P) ≥ 1 and Q value < 0.05, resulting in the identification of a set of differentially expressed genes (DEGs) between Cashmere goats and normal goats.

**Figure 8:**
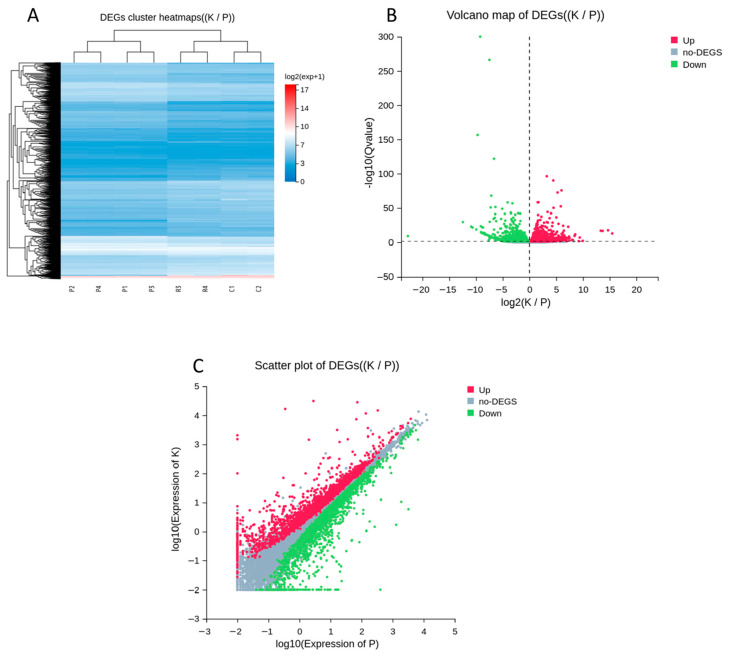
Differential expression analysis of hair follicle–related genes between Cashmere goats (K) and normal goats (P). (**A**) Hierarchical clustering heatmap of differentially expressed genes (DEGs) based on normalized log_2_ (expression + 1) values, demonstrating expression patterns across K and P samples. (**B**) Volcano plot depicting DEGs between K and P groups, where red and green dots indicate significantly upregulated and downregulated genes, respectively, as determined by log_2_ (K vs. P) ≥ 1 and Q-value < 0.05. (**C**) Scatter plot comparing log_10_-transformed gene expression levels between K and P, distinguishing upregulated, downregulated, and non-differentially expressed genes.

Hierarchical clustering analysis of DEGs revealed distinct expression patterns that separated Cashmere goat samples from normal goat samples (Figure 8A). Numerous genes showed coordinated upregulation or downregulation in the Cashmere group, indicating systematic transcriptional reprogramming in hair follicle biology. The volcano plot illustrated the magnitude and significance of differential expression, with red and green points representing 1543 statistically significant DEGs (Q value < 0.05, log_2_ FC ≥ 1), including 721 upregulated and 822 downregulated genes in Cashmere goats compared to normal goats (Figure 8B). Additionally, a scatter plot of log_10_-transformed expression levels showed the overall distribution of DEGs and confirmed consistent expression trends between the two groups (Figure 8C).

### 3.6. Comparative Analysis of DEGs Across Multiple Contrasts

**Figure 9:**
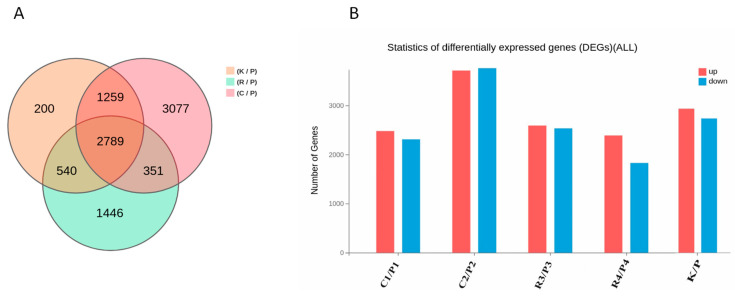
(**A**) The Venn diagram displays the overlap and distinct sets of differentially expressed genes (DEGs) identified in three pairwise comparisons: K vs. P, C vs. P, and R vs. P. The numbers represent genes that are uniquely or commonly differentially expressed among these comparisons. (**B**) The statistical distribution of DEGs is shown for each pairwise comparison between cashmere and normal goats. Bar plots indicate the number of upregulated (red) and downregulated (blue) genes identified in C1 vs. P1, C2 vs. P2, R3 vs. P3, R4 vs. P4, and the combined K vs. P comparison, using the criteria of absolute log_2_ fold change of at least 1 and Q value less than 0.05.

To further analyze transcriptional differences, differentially expressed genes (DEGs) were compared across the pairwise contrasts K vs. P, C vs. P, and R vs. P (Figure 9A). The Venn diagram illustrates the overlap of DEGs among these three comparisons between Cashmere and normal goats. A total of 2789 genes were commonly differentially expressed across K vs. P, C vs. P, and R vs. P, suggesting a shared transcriptional response that distinguishes Cashmere goats from normal goats. Pairwise overlaps identified 1259 genes shared between K vs. P and C vs. P, 540 between K vs. P and R vs. P, and 351 between C vs. P and R vs. P. Each comparison also identified unique DEGs: 200 genes specific to K vs. P, 3077 genes specific to C vs. P, and 1446 genes specific to R vs. P.

Differential expression analysis revealed a substantial number of differentially expressed genes (DEGs) across all pairwise comparisons between Cashmere and normal goats (Figure 9B). In the C1 vs. P1 comparison, approximately 2500 genes were upregulated, and 2300 were downregulated. The C2 vs. P2 comparison showed the highest DEG count, with about 3600 upregulated and 3700 downregulated genes. Comparisons R3 vs. P3 and R4 vs. P4 exhibited moderate but consistent differential expression, with approximately 2600 upregulated and 2500 downregulated genes, and 2400 upregulated and 1800 downregulated genes, respectively. In the overall K vs. P comparison, around 2900 genes were upregulated, and 2700 were downregulated. The comparable numbers of upregulated and downregulated genes across comparisons indicate extensive transcriptomic remodeling associated with the Cashmere phenotype.

### 3.7. Functional Enrichment Analysis of DEGs

Gene Ontology (GO) and Kyoto Encyclopedia of Genes and Genomes (KEGG) enrichment analyses were conducted to investigate the biological functions and pathways associated with the identified differentially expressed genes (DEGs).

**Figure 10:**
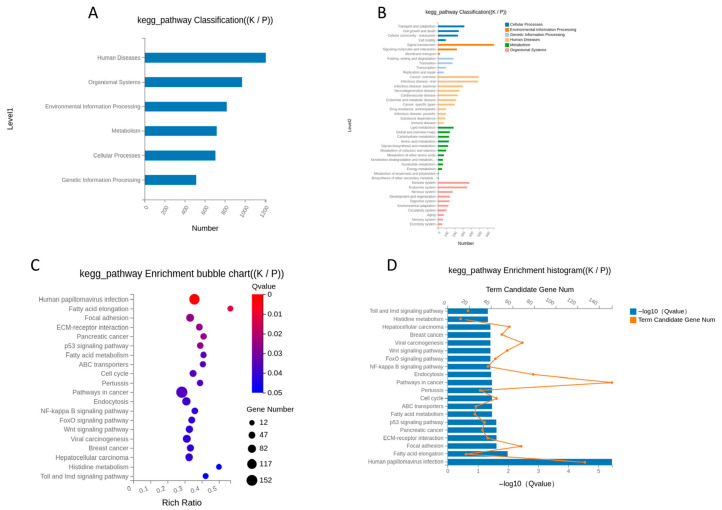
KEGG pathway enrichment analysis of DEGs. (**A**) KEGG Pathway Classification; (**B**) KEGG Pathway Classification Detail; (**C**) KEGG Pathway Enrichment Bubble Chart, significantly enriched KEGG pathways among genes upregulated (red) and downregulated (blue) in Cashmere goats. Key enriched pathways related to hair follicle biology, such as Wnt signaling and extracellular matrix (ECM) interaction, are highlighted; (**D**) KEGG Pathway Enrichment Histogram.

KEGG pathway enrichment analysis indicated that differentially expressed genes were significantly enriched for pathways related to hair follicle development, cell proliferation, and signal transduction, including the Wnt signaling pathway, MAPK signaling pathway, and PI3K-Akt signaling pathway (Figure 10). Pathways directly associated with skin development and hair growth were also significantly enriched (Q value < 0.05), underscoring their potential roles in Cashmere fiber formation.

**Figure 11:**
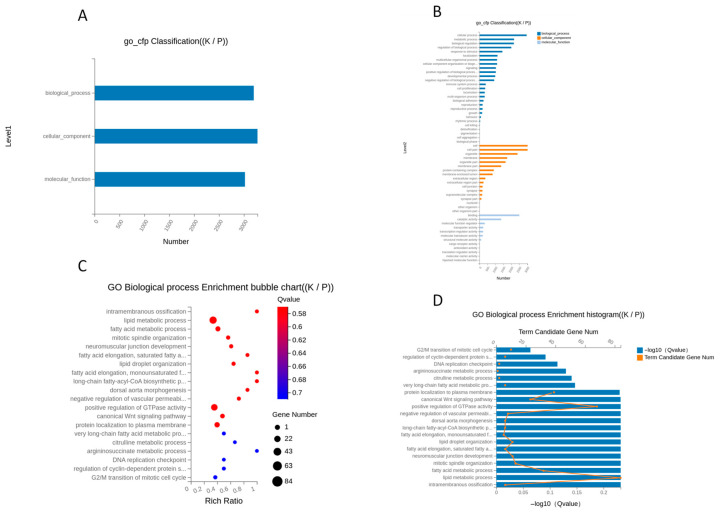
Gene Ontology (GO) enrichment analysis of differentially expressed genes (DEGs). The analysis identifies significant enrichment of DEGs within the three GO categories: Biological Process (BP), Cellular Component (CC), and Molecular Function (MF). Terms related to skin development, epidermal differentiation, and keratinization are notably overrepresented. (**A**) GO Term Classification Summary (K vs. P); (**B**) Detailed GO Term Classification (K vs. P); (**C**) GO Biological Process Enrichment Bubble Chart (K vs. P); (**D**) GO Biological Process Enrichment Histogram (K vs. P).

GO functional enrichment analysis further classified DEGs into biological process, cellular component, and molecular function categories (Figure 11). Enriched biological processes included epidermis development, hair follicle morphogenesis, keratinocyte differentiation, and regulation of cell proliferation. At the cellular component level, DEGs were primarily associated with the extracellular matrix, plasma membrane, and cytoskeletal structures, while molecular function analysis highlighted binding activities and transcriptional regulation.

**Figure 12:**
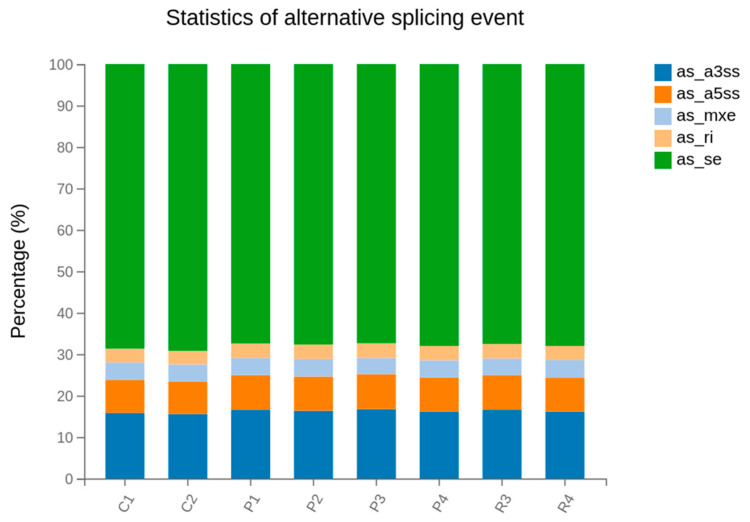
Distribution of alternative splicing event types in Cashmere and normal goat skin transcriptomes. The stacked bar plots present the percentages of five major alternative splicing events, A3SS, A5SS, MXE, RI, and SE, across individual samples.

Alternative splicing (AS) events were systematically analyzed to assess post-transcriptional regulatory complexity in the skin transcriptomes of Cashmere and normal goats. Five major types of AS events were identified in all samples: alternative 3′ splice site (A3SS), alternative 5′ splice site (A5SS), mutually exclusive exons (MXE), intron retention (RI), and exon skipping (SE) (Figure 12).

Exon skipping (SE) was the predominant alternative splicing (AS) event across all samples, accounting for approximately 65 to 70 per cent of total splicing events. This finding suggests that exon inclusion or exclusion is the primary mode of post-transcriptional regulation in goat skin tissue. Alternative 3′ splice site (A3SS) and alternative 5′ splice site (A5SS) events were the next most abundant categories, together contributing roughly 25 to 30 per cent of total AS events. In contrast, mutually exclusive exons (MXE) and intron retention (RI) occurred at much lower frequencies, each representing less than 5 per cent of total splicing events.

## 4. Discussion

This study compares the skin transcriptomes of Cashmere and normal goats, showing major differences in gene expression, pathway activity, and post-transcriptional regulation linked to hair follicle biology. Using high-quality RNA-seq data and multiple analyses, we identify potential molecular mechanisms underlying the Cashmere trait, particularly those related to secondary hair follicle development and fiber formation [16,31,32,33,34].

The high correlation within each group and the clustering of Cashmere and normal goats along the first principal component indicate that fleece type primarily drives transcriptional differences in the skin. This agrees with recent studies showing that hair follicle type and cycle stage strongly affect gene expression in goat skin. Similar overall expression profiles across individuals suggest that group differences arise from specific gene sets being co-regulated, rather than from broad changes in transcription or sequencing depth. This stable background makes it easier to interpret differential expression as meaningful, especially for pathways involved in follicle development and cashmere fiber traits [16,31,32,33].

Finding 1543 significant DEGs between Cashmere and normal goats, with similar numbers of up and downregulated genes, indicates major changes in gene expression linked to the Cashmere trait. The clear separation of Cashmere and normal samples in the heatmaps and the wide range of fold changes in the plots suggest that both the activation and inactivation of gene networks shape these differences. Other studies also report numerous DEGs associated with hair follicle development and fiber quality, supporting the idea that cashmere traits are controlled by multiple genes and complex networks. Many DEGs are shared across comparisons, suggesting a core gene signature that sets Cashmere goats apart and may reflect stable genetic or epigenetic differences, rather than age effects [31,33,34,35].

Each comparison also identified numerous unique DEGs, indicating that developmental stage, age, or individual differences affect the main Cashmere-related gene-expression program. Other studies have reported similar changes with age and hair cycle stage, with gene expression and pathway activity shifting during the anagen, catagen, and telogen phases. These findings suggest that the Cashmere trait results from both breed-specific regulation and changes in follicle biology over time, and that both shared and stage-specific DEGs matter for understanding cashmere fiber traits [16,31,33,34,36].

Functional enrichment analysis showed that many DEGs are part of pathways and processes directly linked to hair follicle development, skin cell differentiation, and extracellular matrix organization. KEGG analysis identified key signaling pathways, including Wnt, MAPK, and PI3K-Akt, that regulate hair follicle growth, stem cell activity, and hair cycle changes in goats and other mammals. Recent studies confirm that the PI3K-Akt and Wnt pathways are central to secondary follicle development, communication between skin layers, and the control of cashmere growth and quality, underscoring their importance here [31,33,36,37,38]. Other research also shows that Wnt signalling affects breed-specific follicle activity and coat type, underscoring its broad role in texture- and density-related traits in goats and other animals [39].

In addition to pathway enrichment analysis, several representative differentially expressed genes (DEGs) with potential relevance to skin biology and hair follicle function were identified. Notably, CAV1 participates in membrane signalling and cell proliferation associated with skin structure, and its dysregulation has been linked to altered epidermal physiology and follicular responses [40]. IL10 is a key anti-inflammatory cytokine that regulates local immune responses and tissue remodelling in the skin [41]. LPL, a key enzyme in lipid metabolism, may influence energy supply and follicular metabolism relevant to hair cycling [42]. Apoptosis-associated BAX has been implicated in hair follicle regression and programmed cell death during the hair cycle [43]. Particularly, regulators of hair cycle control and follicle morphogenesis, including FGF5, LHX2, SOX9, and VDR, were differentially expressed. FGF5 is a well-known negative regulator of hair elongation and promotes the anagen-to-catagen transition, with loss-of-function variants producing long-hair phenotypes in multiple mammals and in humans [44]. LHX2 is required for maintaining hair follicle stem cell character and proper follicle morphogenesis, while SOX9 is essential for outer root sheath differentiation and establishment of the hair stem cell compartment [45]. VDR signalling is also critical for postnatal hair cycling and follicle homeostasis, and VDR disruption causes alopecia phenotypes in animal models and humans [46].

In goats, MAPK activation affects hair follicle growth by interacting with growth factor receptors, such as those in the FGF and FGFR axes, thereby supporting its role in hair follicle cycling and development [47,48]. This suggests that changes in MAPK signalling in Cashmere goats may help create unique follicle patterns that support secondary hair growth.

GO enrichment also highlighted terms such as skin development, hair follicle formation, keratinocyte differentiation, cell growth control, and components of the extracellular matrix and the cytoskeleton. This shows that both skin and connective tissue cells contribute to the observed differences in gene expression. Single-cell studies in cashmere goats have identified distinct keratinocyte types and dermal papilla subtypes with unique gene signatures, underscoring the complexity of cell-specific roles in follicle development. The agreement between these GO and KEGG results and earlier studies suggests that the DEGs identified here are part of known follicle regulatory networks and could be promising candidates for further research on cashmere fiber formation [16,31,32,33].

Gene fusion events were rare and did not show group-specific patterns, suggesting that large structural changes in transcripts are unlikely to be the main cause of differences between Cashmere and normal goats in this study. This aligns with other goat transcriptome studies, which also report a few recurring fusions in skin compared with other tissues or diseases. On the other hand, alternative splicing was common, with exon skipping as the main type, accounting for most splicing events in the samples [34,49].

A recent study using both second- and third-generation transcriptome analysis in goat skin also found that exon skipping is the most common splicing pattern. This suggests that controlling which exons are included or skipped is important for creating diversity in follicle-related genes. Similar splicing events in both Cashmere and normal goats indicate that general splicing processes are shared, but it is still possible that certain gene isoforms are more common in one group and affect fiber traits. Future research using isoform-level analysis and long-read sequencing could determine whether specific splice variants in keratin, signalling, or extracellular matrix genes are more common in Cashmere follicles and influence fiber features such as diameter, length, or structure [32,34,49].

## 5. Conclusions

This study provides a comparative transcriptomic characterization of skin tissues from Inner Mongolian cashmere goats and normal goats, revealing clear molecular distinctions associated with cashmere fiber production. High-quality RNA-seq data and robust statistical analyses demonstrated pronounced transcriptional divergence between fleece types, supporting the reliability of the identified differentially expressed genes and downstream functional interpretations. The detected DEGs were primarily enriched in biological processes related to hair follicle morphogenesis, epidermal differentiation, cellular proliferation, and extracellular matrix organization, highlighting coordinated regulation of epidermal and dermal components during secondary hair follicle activity.

Consistent with established mechanisms of follicle development and cycling, key signaling pathways including Wnt, MAPK, and PI3K–Akt were significantly enriched, and several representative regulators of hair follicle development and hair cycle control were differentially expressed. In addition to transcriptional regulation, alternative splicing, particularly exon skipping, emerged as an important transcriptome-level regulatory feature in goat skin, suggesting an additional layer of molecular control contributing to follicle function and fibre traits.

Collectively, these findings refine the molecular framework underlying secondary hair follicle biology and cashmere fiber formation and provide candidate genes and pathways for future functional validation and molecular breeding strategies aimed at improving economically important fleece traits in cashmere goats.

## Data Availability

All data needed to evaluate the conclusions in this paper are present either in the main text or the Supplementary Materials.

## Supplementary Materials

The following supporting information can be downloaded at: https://www.mdpi.com/article/10.3390/ani16060927/s1. Table S1: Summary of RNA-seq data quality metrics, including total reads, clean read numbers, clean read ratios, and base quality scores (Q20 and Q30) for all cashmere and normal goat skin samples. Table S2: Mapping statistics of clean reads aligned to the goat reference genome, including total mapping rate and uniquely mapped reads, reflecting overall alignment efficiency. Table S3: Mapping statistics of clean reads aligned to the reference gene set, showing gene-level alignment rates and transcript coverage distribution across samples. Table S4: Complete list of differentially expressed genes (DEGs) identified in the K vs. P comparison, including gene ID, gene name, log_2_ fold change, Q value, and regulation direction. Table S5: Complete list of differentially expressed genes (DEGs) identified in the C vs. P comparison. Table S6: Complete list of differentially expressed genes (DEGs) identified in the R vs. P comparison.

## References

[1] Waldron S., Brown C., Komarek A.M. (2014). The Chinese Cashmere Industry: A Global Value Chain Analysis. Dev. Policy Rev..

[2] Wang F.H., Gong G., Yan X.C., Zhang L.T., Zhang F.T., Liu H.F., Lv Q., Wang R.J., Zhang Y.J., Wang Z.X. (2021). Genome-wide association study of fleece traits in Inner Mongolia Cashmere goats. Anim. Genet..

[3] Stenn K.S., Paus R. (2001). Controls of Hair Follicle Cycling. Physiol. Rev..

[4] Oh J.W., Kloepper J., Langan E.A., Kim Y., Yeo J., Kim M.J., Hsi T.C., Rose C., Yoon G.S., Lee S.J. (2016). A Guide to Studying Human Hair Follicle Cycling In Vivo. J. Investig. Dermatol..

[5] Schneider M.R., Schmidt-Ullrich R., Paus R. (2009). The Hair Follicle as a Dynamic Miniorgan. Curr. Biol..

[6] Han W., Yang F., Wu Z., Guo F., Zhang J., Hai E., Shang F., Su R., Wang R., Wang Z. (2020). Inner Mongolian Cashmere Goat Secondary Follicle Development Regulation Research Based on mRNA-miRNA Co-analysis. Sci. Rep..

[7] Zhang Y., Wu K., Wang L., Wang Z., Han W., Chen D., Wei Y., Su R., Wang R., Liu Z. (2020). Comparative study on seasonal hair follicle cycling by analysis of the transcriptomes from cashmere and milk goats. Genomics.

[8] Yang M., Song S., Dong K., Chen X., Liu X., Rouzi M., Zhao Q., He X., Pu Y., Guan W. (2017). Skin transcriptome reveals the intrinsic molecular mechanisms underlying hair follicle cycling in Cashmere goats under natural and shortened photoperiod conditions. Sci. Rep..

[9] Lin X., Zhu L., He J. (2022). Morphogenesis, Growth Cycle and Molecular Regulation of Hair Follicles. Front. Cell Dev. Biol..

[10] Hébert J.M., Rosenquist T., Götz J., Martin G.R. (1994). FGF5 as a regulator of the hair growth cycle: Evidence from targeted and spontaneous mutations. Cell.

[11] Kljuic A., Bazzi H., Sundberg J.P., Martinez-Mir A., O’SHaughnessy R., Mahoney M.G., Levy M., Montagutelli X., Ahmad W., Aita V.M. (2003). Desmoglein 4 in Hair Follicle Differentiation and Epidermal Adhesion. Cell.

[12] Geng R., Yuan C., Chen Y. (2013). Exploring Differentially Expressed Genes by RNA-Seq in Cashmere Goat (*Capra hircus*) Skin during Hair Follicle Development and Cycling. PLoS ONE.

[13] Nocelli C., Cappelli K., Capomaccio S., Pascucci L., Mercati F., Pazzaglia I., Mecocci S., Antonini M., Renieri C. (2020). Shedding light on cashmere goat hair follicle biology: From morphology analyses to transcriptomic landascape. BMC Genom..

[14] Wang S., Ge W., Luo Z., Guo Y., Jiao B., Qu L., Zhang Z., Wang X. (2016). Comparative transcriptome analysis of fetal skin reveals key genes related to hair follicle morphogenesis in cashmere goats. PLoS ONE.

[15] Yang F., Li R., Zhao C., Che T., Guo J., Xie Y., Wang Z., Li J., Liu Z. (2022). Single-cell sequencing reveals the new existence form of dermal papilla cells in the hair follicle regeneration of cashmere goats. Genomics.

[16] Ge W., Zhang W., Zhang Y., Zheng Y., Li F., Wang S., Liu J., Tan S., Yan Z., Wang L. (2021). A Single-Cell Transcriptome Atlas of Cashmere Goat Hair Follicle Morphogenesis. Genom. Proteom. Bioinform..

[17] Ma H., Zhang W., Song W.H., Sun P., Jia Z.H. (2012). Effects of tryptophan supplementation on cashmere fiber characteristics, serum tryptophan, and related hormone concentrations in cashmere goats. Domest. Anim. Endocrinol..

[18] Chomczynski P., Sacchi N. (2006). The single-step method of RNA isolation by acid guanidinium thiocyanate–phenol–chloroform extraction: Twenty-something years on. Nat. Protoc..

[19] Desjardins P., Conklin D. (2010). NanoDrop Microvolume Quantitation of Nucleic Acids. J. Vis. Exp..

[20] Parkhomchuk D., Borodina T., Amstislavskiy V., Banaru M., Hallen L., Krobitsch S., Lehrach H., Soldatov A. (2009). Transcriptome analysis by strand-specific sequencing of complementary DNA. Nucleic Acids Res..

[21] Drmanac R., Sparks A.B., Callow M.J., Halpern A.L., Burns N.L., Kermani B.G., Carnevali P., Nazarenko I., Nilsen G.B., Yeung G. (2010). Human Genome Sequencing Using Unchained Base Reads on Self-Assembling DNA Nanoarrays. Science.

[22] Li R., Li Y., Kristiansen K., Wang J. (2008). SOAP: Short oligonucleotide alignment program. Bioinformatics.

[23] Kim D., Langmead B., Salzberg S.L. (2015). HISAT: A fast spliced aligner with low memory requirements. Nat. Methods.

[24] Langmead B., Salzberg S.L. (2012). Fast gapped-read alignment with Bowtie 2. Nat. Methods.

[25] Li B., Dewey C.N. (2011). RSEM: Accurate transcript quantification from RNA-Seq data with or without a reference genome. BMC Bioinform..

[26] Mao J., He J., Ren Y., Li X., Yang C., Liu G., Zhang G., Wei C., Zhang W., Wang M. (2025). Mining of candidate genes related to prolificacy in Jining grey goats using transcriptomics. BMC Genom..

[27] Love M.I., Huber W., Anders S. (2014). Moderated estimation of fold change and dispersion for RNA-seq data with DESeq2. Genome Biol..

[28] Wang L., Feng Z., Wang X., Wang X., Zhang X. (2010). DEGseq: An R package for identifying differentially expressed genes from RNA-seq data. Bioinformatics.

[29] Audic S., Claverie J.-M. (1997). The Significance of Digital Gene Expression Profiles. Genome Res..

[30] Benelli M., Pescucci C., Marseglia G., Severgnini M., Torricelli F., Magi A. (2012). Discovering chimeric transcripts in paired-end RNA-seq data by using EricScript. Bioinformatics.

[31] Shen S., Park J.W., Lu Z., Lin L., Henry M.D., Wu Y.N., Zhou Q., Xing Y. (2014). rMATS: Robust and flexible detection of differential alternative splicing from replicate RNA-Seq data. Proc. Natl. Acad. Sci. USA.

[32] Qin Z., Sun X., Sun L., Yu M., Jiang H. (2025). Transcriptome sequencing reveals the key genes associated with hair follicle development in Qianhua Mutton Merino. Front. Vet. Sci..

[33] Zhao P., Guo H., Han J., Wang Z., Xue Y., Zhang L. (2025). Transcriptome sequencing reveals the expression profiles of lncRNAs and mRNAs in goat skin tissues with different types of wool coats. Sci. Rep..

[34] Gong G., Bi S., Liang X., Ao Y., Xu F., Sulaiman Y. (2025). Molecular mechanisms underlying cashmere quality differences between Jiangnan cashmere goats and Changthangi pashmina goats. Front. Vet. Sci..

[35] Zhao J., Zhang J., Chhen Z., Xiao M., Zhao Y. (2025). Whole-transcriptome RNA sequencing reveals global expression dynamics and ceRNA regulatory networks related to hair follicle development and melanogenesis in goats. Anim. Biosci..

[36] Gong G., Fan Y., Yan X., Li W., Yan X., Liu H., Zhang L., Su Y., Zhang J., Jiang W. (2022). Identification of Genes Related to Hair Follicle Cycle Development in Inner Mongolia Cashmere Goat by WGCNA. Front. Vet. Sci..

[37] Gong W., Liu J., Mu Q., Chahaer T., Liu J., Ding W., Bou T., Wu Z., Zhao Y. (2024). Melatonin promotes proliferation of Inner Mongolia cashmere goat hair follicle papilla cells through Wnt10b. Genomics.

[38] Zhou B., Xi H., Yu Y., Li J., Su R., Lv Q., Zhang Y., Wang R., Wang Z. (2025). Comparative analysis of skin transcriptome reveals differences of cashmere fineness in different body parts of Inner Mongolia cashmere goats. Anim. Biosci..

[39] Xie L., Li C., Tian J., Fu S., Su R., Feng C., Chen M. (2025). Integrated transcriptomic and proteomic analysis reveals molecular mechanisms of melatonin-regulated hair follicle growth in cashmere goats and comparative insights across goat breeds. BMC Genom..

[40] Takamura N., Yamaguchi Y. (2022). Involvement of caveolin-1 in skin diseases. Front. Immunol..

[41] King A., Balaji S., Le L.D., Crombleholme T.M., Keswani S.G. (2014). Regenerative Wound Healing: The Role of Interleukin-10. Adv. Wound Care (New Rochelle).

[42] Li Y., He P.-P., Zhang D.-W., Zheng X.-L., Cayabyab F.S., Yin W.-D., Tang C.-K. (2014). Lipoprotein lipase: From gene to atherosclerosis. Atherosclerosis.

[43] Wang W., Wang H., Long Y., Li Z., Li J. (2023). Controlling Hair Loss by Regulating Apoptosis in Hair Follicles: A Comprehensive Overview. Biomolecules.

[44] Higgins C.A., Petukhova L., Harel S., Ho Y.Y., Drill E., Shapiro L., Wajid M., Christiano A.M. (2014). FGF5 is a crucial regulator of hair length in humans. Proc. Natl. Acad. Sci. USA.

[45] Rhee H., Polak L., Fuchs E. (2006). Lhx2 Maintains Stem Cell Character in Hair Follicles. Science.

[46] Demay M.B., MacDonald P.N., Skorija K., Dowd D.R., Cianferotti L., Cox M. (2007). Role of the vitamin D receptor in hair follicle biology. J. Steroid Biochem. Mol. Biol..

[47] Xi M., Jiang J., Wang B., Wang Y., Di M., Cong Y., Zhang R. (2025). Alterations in Methionine Cycle and Wnt/MAPK Signaling Associated with HMBi-Induced Cashmere Growth in Goats. Int. J. Mol. Sci..

[48] Su R., Gong G., Zhang L., Yan X., Wang F., Zhang L., Qiao X., Li X., Li J. (2020). Screening the key genes of hair follicle growth cycle in Inner Mongolian Cashmere goat based on RNA sequencing. Arch. Anim. Breed..

[49] Yang X., Zheng L., Huo J., Hu W., Liu B., Fan Q., Zheng W., Wang Q. (2024). Combined Analysis of Second- and Third-Generation Transcriptome Sequencing for Gene Characteristics and Identification of Key Splicing Variants in Wound Healing of Ganxi Goat Skin. Animals.

